# Using Micro-Molding and Stamping to Fabricate Conductive Polydimethylsiloxane-Based Flexible High-Sensitivity Strain Gauges

**DOI:** 10.3390/s18020618

**Published:** 2018-02-18

**Authors:** Chi-Jui Han, Hsuan-Ping Chiang, Yun-Chien Cheng

**Affiliations:** Department of Mechanical Engineering, National Chiao Tung University, Hsinchu 300, Taiwan; dragon199357@gmail.com (C.-J.H.); b98502141@ntu.edu.tw (H.-P.C.)

**Keywords:** conductive PDMS, strain gauge, carbon particle, stamping-process, gauge factor, piezoresistance

## Abstract

In this study, polydimethylsiloxane (PDMS) and conductive carbon nanoparticles were combined to fabricate a conductive elastomer PDMS (CPDMS). A high sensitive and flexible CPDMS strain sensor is fabricated by using stamping-process based micro patterning. Compared with conventional sensors, flexible strain sensors are more suitable for medical applications but are usually fabricated by photolithography, which suffers from a large number of steps and difficult mass production. Hence, we fabricated flexible strain sensors using a stamping-process with fewer processes than photolithography. The piezoresistive coefficient and sensitivity of the flexible strain sensor were improved by sensor pattern design and thickness change. Micro-patterning is used to fabricate various CPDMS microstructure patterns. The effect of gauge pattern was evaluated with ANSYS simulations. The piezoresistance of the strain gauges was measured and the gauge factor determined. Experimental results show that the piezoresistive coefficient of CPDMS is approximately linear. Gauge factor measurement results show that the gauge factor of a 140.0 μm thick strain gauge with five grids is the highest.

## 1. Introduction

Wearable and skin attachable electronic devices have gained attention in recent years. Such applications are ideal for biomedical microelectromechanical systems (bioMEMS) [1,2,3,4]. Flexible strain sensors can be used in many biomedical applications [5,6]. They can be attached to clothes to detect body motion signals [6,7,8,9], such as buckling, walking, crouching, and jumping. Flexible strain sensors can also be attached to the skin above the vocal cords to detect electrical signals during speech and breathing [10]. 

A conductive elastomer is a composite containing both flexible material and conductive material, which can be applied to fabricate many kinds of sensors, such as temperature [11], pressure [12,13,14,15], and strain sensors [16,17,18,19,20]. These sensors can also be used in biomedical applications, such as blood pulse measurement [21] and as electronic skin [22]. A common way to fabricate a conductive elastomer is by mixing flexible material and conductive material, and then curing the composites by baking. The most commonly used flexible material for conductive elastomer fabrication is polydimethylsiloxane (PDMS) [23,24,25,26,27,28,29,30,31,32,33].

There are many methods to fabricate flexible strain sensors with conductive elastomers, including carbon nanotube growth [16,34,35,36], photolithography [37], and soft lithography. In 2008, Chang et al. fabricated flexible strain sensors by carbon nanotube growth. These flexible strain sensors achieved a high strain resolution of 0.004% and a high piezoresistive gauge factor of 269 [16]. In 2012, Lu et al. fabricated flexible strain sensors by photolithography with carbon nanoparticles, carbon nanotubes, and PDMS [37]. The manufacturing procedure required undergoing photolithography processes twice, resulting in a piezoresistive gauge factor of 29.1. In 2014, Kong et al. fabricated flexible strain sensors by soft lithography micropatterning, using carbon nanoparticles and PDMS [24]. The piezoresistive gauge factor of the sensor was about 1.8–5.5. In summary, most conductive-elastomer based flexible strain sensors were fabricated by photolithography, which can achieve a higher piezoresistive gauge factor (approximately 15–30), but is process intensive and time consuming. Soft lithography fabrication requires less processing than photolithography fabrication, thus it is more cost effective, but flexible strain sensors fabricated by soft lithography can only achieve a lower piezoresistive gauge factor (approximately 1.8–5.5) because of the limitations of the materials used. As a result, soft lithography-based sensors can only be used in applications requiring low piezoresistive gauge factors, such as health-monitoring, mass measurement, and pressure sensing.

In this study, different pattern of flexible strain sensors was made by a stamping-process with conductive PDMS (CPDMS) elastomer, trying to improve the gauge factor by patterning. The PDMS stamp was made of micromolding. The goal is to provide high gauge-factor strain sensors without the intensive processes required by photolithography. First we measured the Young’s modulus and piezoresistive gauge factors of the CPDMS strain sensors. Relevant parameters, such as length and thickness, of the sensor were modified to enhance the gauge factor. The thickness of the resulting CPDMS strain sensors was measured with an optical microscope.

## 2. Materials and Methods

### 2.1. Fabrication of Conductive Elastomer 

Carbon particles (Colour Black FW 200, Palmer Holland, North Olmsted, OH, USA) with 10 nm diameter were used. Reference [38] 5 wt % carbon particles [39] and PDMS (SYLGARD 184 A, U&I Bio-Tech, Seoul, Korea) with mixing ratio of 10:1 (A agent:B agent) were mixed with heptane. Then the mixed matrix was stirred till the heptane was completely volatilized so the carbon particles can be fully dispersed in the PDMS matrix. The particle-PDMS matrix was poured into a mold or onto silicon wafers (400 µm thick; Semiconductor Wafer, Hsinchu, Taiwan) for the stamping process. Finally, the matrix was degassed and cured at 70 °C in a vacuum oven (DOV-30, Yeong-Shin, Hsinchu, Taiwan) for one hour.

### 2.2. Fabrication of CPDMS Strain Sensor 

**Figure 1:**
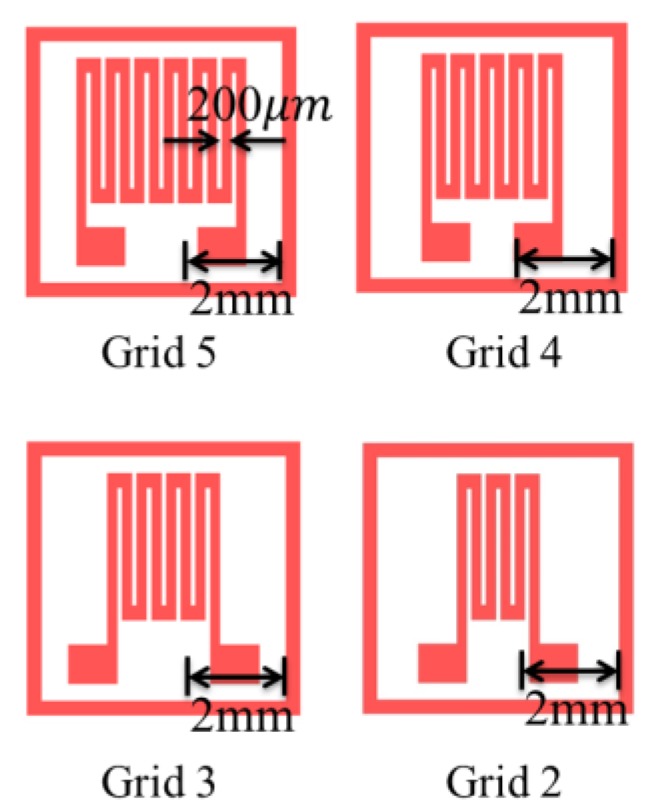
Mask patterns for strain sensor fabrication.

Silicon molds of designed patterns were made by photolithography [40] Grid patterns included patterns with two, three, four, and five grids (Figure 1). 

**Figure 2:**
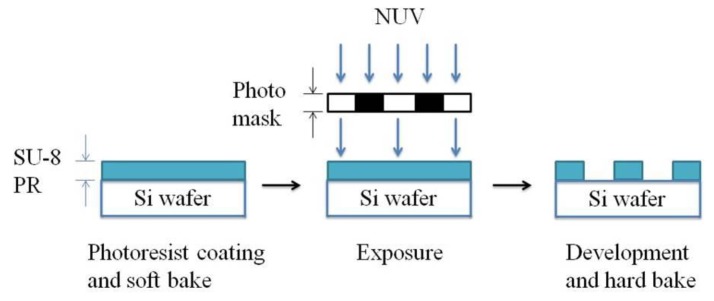
Mold fabrication process by photolithography.

**Figure 3:**
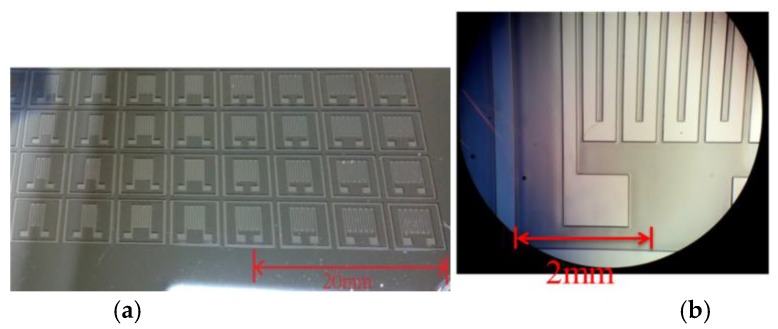
(**a**) Silicon mold and (**b**) optical microscope image of mold for strain sensor fabrication.

Patterns with more grids increase the length of conductive elastomers. The strain and resistance relations of the CPDMS strain sensors with different patterns were measured. We used SU8 (SU8-2025, TELTEC Semiconductor Pacific, Hsinchu, Taiwan) as the photoresist in strain sensor mold fabrication. Parameters used during fabrication are: rotation speed of 1500 rpm, exposure time of 4.5 s, and development time of 9 min. The mold development process with photolithography is shown in Figure 2. The mold fabrication results are shown in Figure 3.

**Figure 4:**
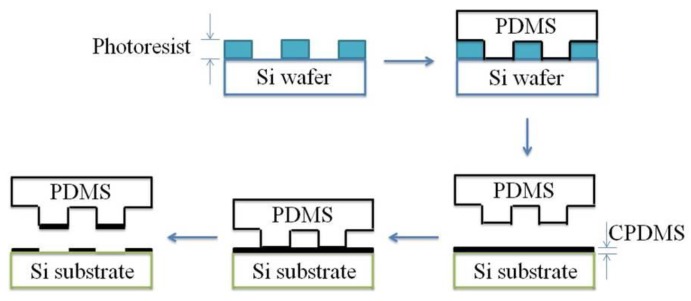
Micro-molding and stamping (micropatterning) fabrication process.

Micropatterning was applied to make CPDMS strain sensors. The fabrication process is shown in Figure 4. First PDMS with a mixing ratio of 10:1 was poured into the silicon mold, degassed in the vacuum oven, and cured at 70 °C. The cured PDMS was peeled off and used as a “stamp”. The PDMS stamp was pressed onto CPDMS mixture on silicon wafers, and then the CPDMS was transferred onto the stamp. After being cured, CPDMS elastomers with different patterns were made and can be used as strain gauge sensors.

### 2.3. Measuring the Piezoresistance of the CPDMS Elastomer

**Figure 5:**
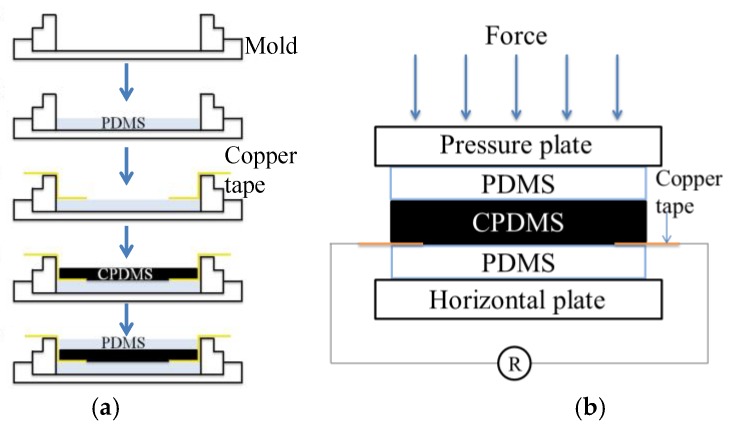
Piezoresistance measurement. (**a**) Preparation and (**b**) CPDMS resistance measurement under different pressure.

The resistance measurement of CPDMS under different pressures is shown in Figure 5. The CPDMS bulk was protected with two PDMS layers. The sandwich structure was made by curing PDMS, CPDMS, and PDMS layers sequentially, and a conductive copper tape was inserted during curing for resistance measurement (Figure 5a). A universal testing machine (HT-9102, Hung Ta Instrument, Taichung, Taiwan) compressed the CPDMS elastomer in the vertical direction, with a resistance meter (DM-3000, HILA, Taipei, Taiwan) attached on the conductive copper tape (Figure 5b). For electrical conditioning, the carbon particle and PDMS ratio was varied till the measured initial resistances can be measured and are reproducible (*n* > 3, data not shown). Also, the resistance of measurement was calibrated till the initial resistances of CPDMS were identical. When pressure was exerted by the universal testing machine, we recorded the force, movement, and electric resistance to calculate piezoresistance. 

### 2.4. Measuring Piezoresistance of the CPDMS Strain Sensor 

**Figure 6:**
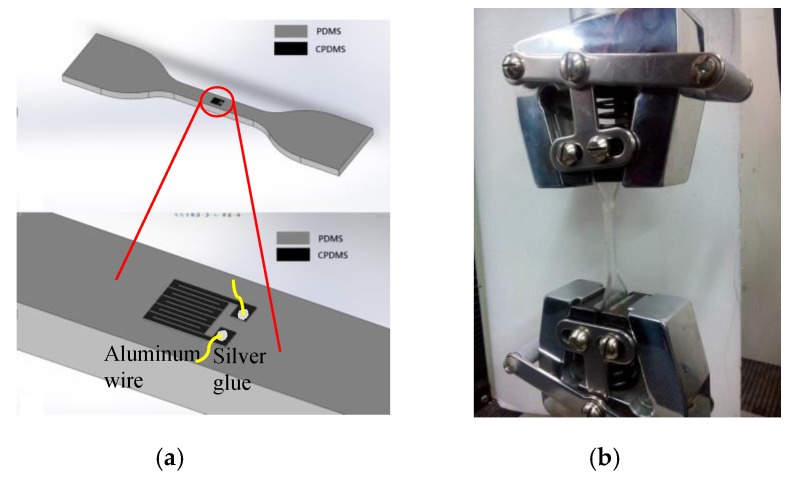
Piezoresistance measurement of (**a**) CPDMS attached on a strain gauge with (**b**) universal testing machine.

The CPDMS strain sensor was fixed on the PDMS substrate as shown in Figure 6a. On the two pads of the CPDMS strain sensor, aluminum wires were fixed with silver glue. PDMS substrate was stretched with the HT-9102 universal testing machine (Figure 6b) and the resistance change of CPDMS strain sensor was measured with the resistance meter.

Under the same initial and boundary condition, PDMS and PDMS strain gauge yielded the same results in the stress-strain simulation, so this method could determine the piezoresistance of the CPDMS strain sensors. With the displacements measurement from the universal testing machine and resistance value, the piezoresistance and the gauge factor of the CPDMS strain sensor could be obtained.

### 2.5. Young’s Modulus Measurement

Young’s modulus measurement of the elastomer was based on the ASTM D412 standard test from the American Society of the International Association for Testing and Materials. The PDMS and CPDMS sample was fixed in the universal testing machine. When the sample was stretched, the computer recorded force and displacement. Stress was calculated according to the formula below (Equation (1)):

(1) σ=F/A

In Equation (1), *σ* is the stress (MPa), *F* is the force (N) and *A* is the cross-sectional area (mm**^2^**) of the sample. Strain was calculated according to the formula below (Equation (2)):

(2) $$\epsilon =(L-L_{0})/L_{0}$$

In Equation (2), *ϵ* is the strain, L is the stretched length (mm) and *L**_0_*** is the initial length (mm). Young’s modulus was calculated according to the formula below (Equation (3)). *E* is the Young’s modulus, *σ* is the stress, and *ϵ* is the strain. The calculated Young’s modulus of PDMS and CPDMS were used for ANSYS stress analysis:

(3) E=σ/ϵ

### 2.6. CPDMS Strain Sensor Simulation

**Figure 7:**
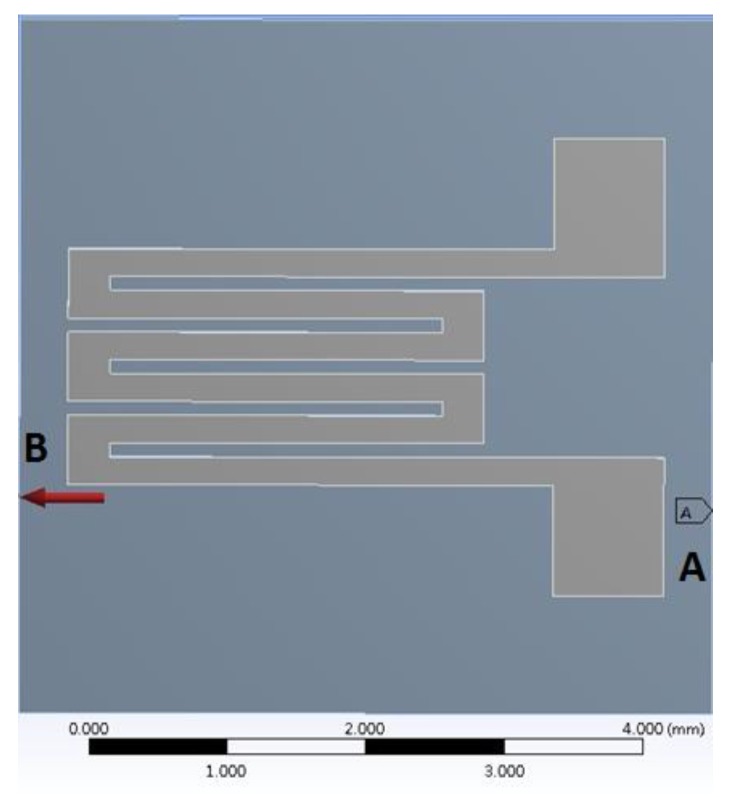
Boundary conditions of PDMS simulation. Right side (**A**) was fixed and the pressure applied on left side (**B**) was 0.2415 MPa.

In this study, the stress-strain analysis of the PDMS substrate and CPDMS strain sensor was simulated with ANSYS Workbench 14.0. For PDMS, the density, Young’s modulus, and Poisson’s ratio used were 965.0 kg/m**^3^**, 410 kPa, and 0.3, respectively; for CPDMS, the density, Young’s modulus and Poisson ratio were 1290.5 kg/m**^3^**, 410 kPa, and 0.3, respectively. Both PDMS and CPDMS Young’s modulus were based on the measurement results mentioned in Section 2.5. The thickness of the PDMS substrate was 1.0 mm and the height of the protruding PDMS structure was 90 μm; the thickness of the CPDMS was measured by optical microscope (DM IL, Leica, Wetzlar, Germany) and was about 30 μm. For boundary conditions, as shown in Figure 7, the right side (A) of the pattern was fixed and 0.2415 MPa tensile stress was exerted on the left side (B). The resulting strain of 0.2415 MPa was 25%.

### 2.7. Durability Test of the CPDMS Strain Sensor

The durability test was performed by stretching the CPDMS gauge sensor multiple times with the universal testing machine while recording the resistance. The CPDMS gauge sensor was stretched to 10% above its initial length and then released repeatedly. The stretching speed was 3 mm/min; amount stretched varied from 0.0 mm to 3.3 mm (10% strain) and releasing amount was from 3.3 mm to 0.0 mm. Initial resistance of the CPDM was first measured. The resistance was measured 5 seconds after every stretching and releasing.

## 3. Results and Discussion

### 3.1. Conductive Elastomer Measurement Results

**Figure 8:**
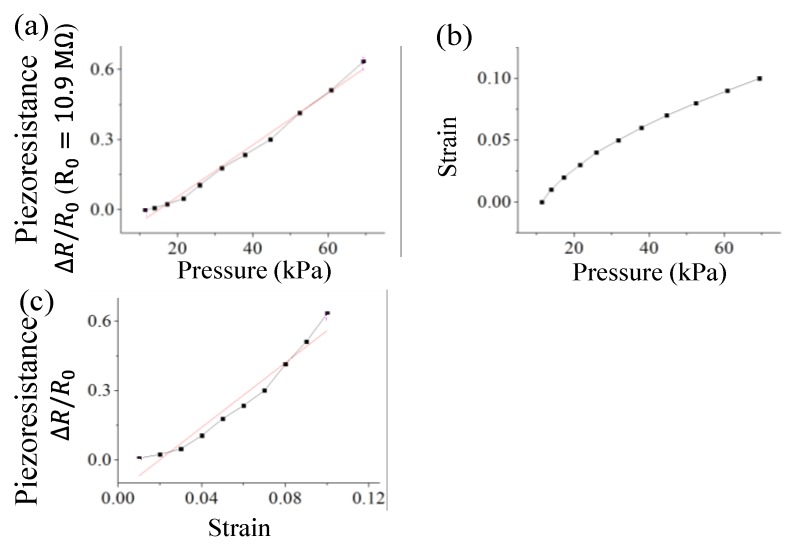
Piezoresistance and strain of CPDMS under different pressure. (**a**) Piezoresistance to pressure; (**b**) Strain to pressure; (**c**) Piezoresistance to strain. The black lines are the measurement results and the red lines are the linear approximation of the measurement results.

The conductive elastomer was put into the universal testing machine and pressure was exerted on the elastomer. The measurement results were shown in Figure 8. As shown in Figure 8a, the resistance of the conductive elastomer rose with the applied pressure. This could be attributed to the transverse elongation of the conductive elastomer, which increased the distance between carbon particles embedded in the conductive elastomers. Figure 8b presents the pressure and strain values (ratio of displacement and sample thickness). Figure 8c is the piezoresistance to strain relation according to Figure 8a,b. The piezoresistance increased linearly with the strain.

### 3.2. CPDMS Strain Sensor Simulation Results

**Figure 9:**
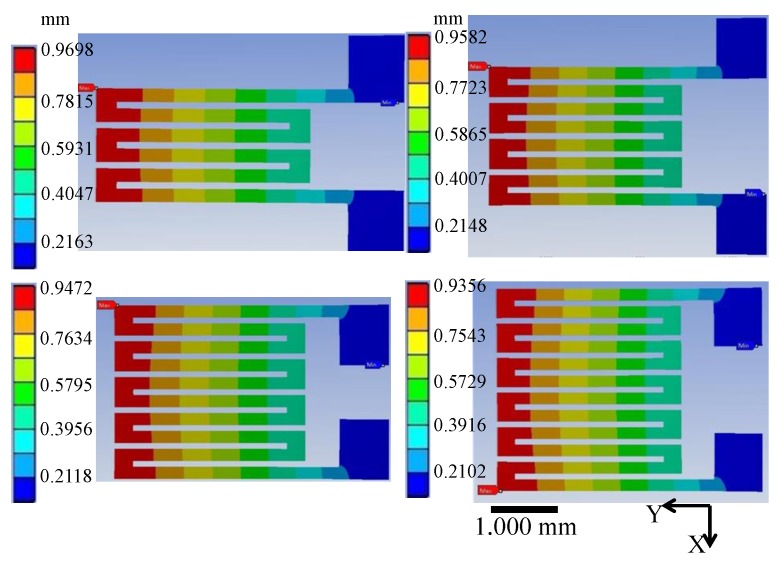
Total stretching and gauge factors of different gauge patterns were simulated with ANSYS.

CPDMS strain sensor simulation results are shown in Figure 9. The stretch on the left side (0.93–0.97) is greater than the stretch on the right side (0.21–0.22). The CPDMS strain sensor with more grids has a larger total strain with the same PDMS substrate strain. Therefore, the corresponding resistance change and gauge factor increase with grid number.

### 3.3. Gauge Factors of CPDMS Strain Sensors with Different Amount of Grids 

**Figure 10:**
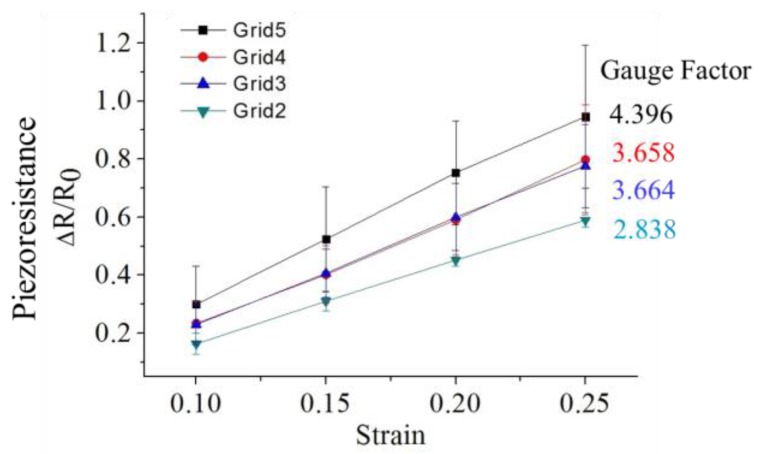
Gauge factors of CPDMS gauge sensor with different grid numbers: squares, gauge factor for the five grid gauge sensor; circles, gauge factor for the four grid gauge sensor; blue triangles, gauge factor for the three grid gauge sensor; green triangles, gauge factor for the two grid gauge sensor.

The resistance change and strain rate of CPDMS strain sensor with different amount of grids was measured. The thickness of the CPDMS was 120.0 μm ± 15.7 μm. The measurement results are shown in Figure 10. When the number of grids increased, the gauge factor increased. This is because sensors with more grids have longer CPDMS structures. Hence, resistance change and gauge factor increased with grid number. This result corresponds to results from simulations. The standard deviation of the measured gauge factor was high sine the CPMDS thickness was difficult to control in the CPDMS “stamping” process. The gauge factors were also divided by the number of sensor squares and the normalized gauge factors were calculated (Table 1). The normalized gauge factors are quite similar. The differences might come from the tolerance of gauge factor and the total strain of the sensors was not exactly proportional to the number of sensor squares.

### 3.4. Gauge Factor of CPDMS Strain Sensors with Different Thicknesses

**Figure 11:**
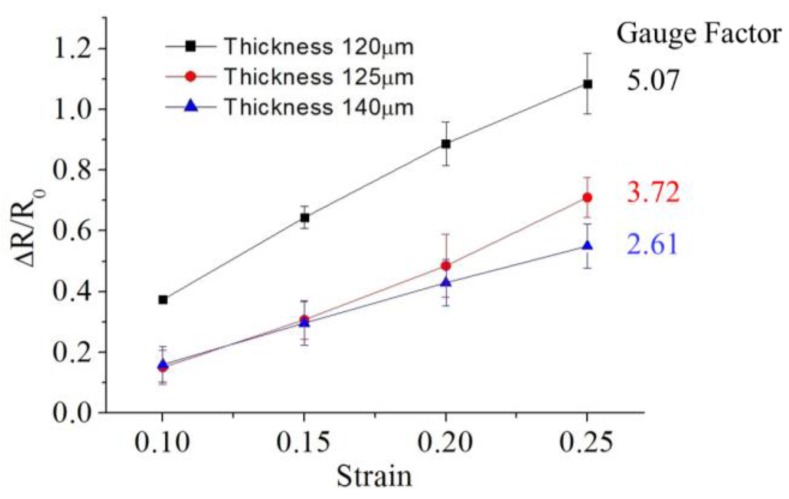
Effects of strain gauge thickness on gauge factor. Squares, gauge factor for the 120 μm gauge sensor; circles, gauge factor for the 125 μm gauge sensor; triangles, gauge factor for the 140 μm gauge sensor.

CPDMS strain sensor with five grids was used for thickness experiments. Sensors with different thicknesses were made by embossing two, four and six times, resulting in thicknesses of 120.0 μm ± 15.7 μm, 125.0 μm ± 19.4 μm, and 140.0 μm ± 13.7 μm, respectively. The measurement results are shown in Figure 11. The gauge factor measured of 120.0 μm thick sensor has the highest gauge factor of about 5.070. This result indicates that the thinner CPDMS strain sensor makes the higher gauge factor. This might be because that for thin CPDMS sensors, the amount of conductive particles is less per unit length. Hence, stretching the sensor makes the resistance change larger than that of thick CPDMS sensors. 

### 3.5. Durability of the CPDMS Strain Sensors 

**Figure 12:**
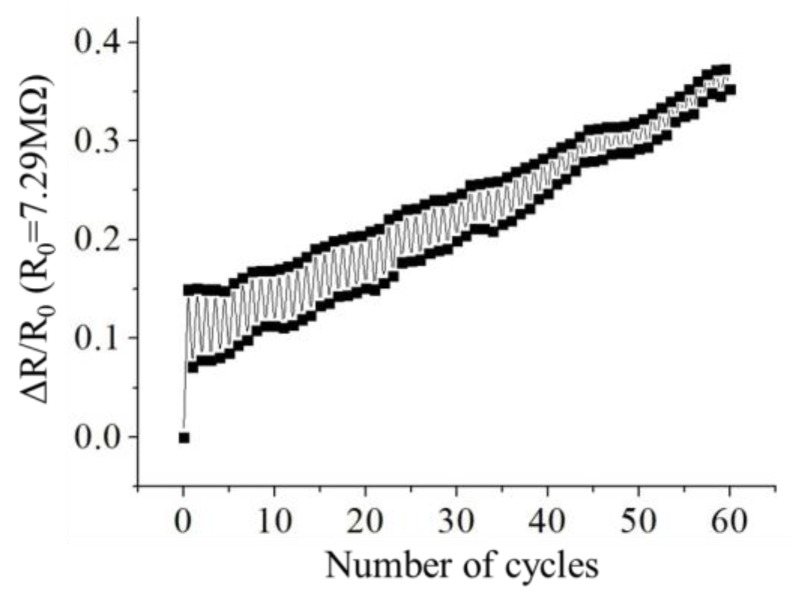
Durability of strain gauge. A 5-grid strain gauge was used. The max strain of stretching was 10% and strain of releasing was 0%.

The CPDMS strain sensor with five grids was used for the durability test, since it has highest gauge factor. The results are shown in Figure 12. The initial resistance of the CPDM strain sensor was 7.29 MΩ. The difference between the first and the second stretch-release cycle might be because of the hysteresis. The increase of the piezoresistance might be because the CPDMS tensile recovery was slow. This would make the distances between carbon particles increase and, hence, piezoresistance increase.

## 4. Conclusions

In this study, CPDMS together with a micro-molding and stamping process was used to fabricate gauge strain sensors rapidly and cost effectively. The effects of different patterns and thickness were also discussed. The results showed that CPDMS resistance increased linearly with compression pressure and the resistance increased 60% at 10% strain. Additional CPDMS strain sensor grids provided higher gauge factors. The five grid pattern attained a gauge factor of 4.396, whereas the two grid pattern measured gauge factor was only 2.838. The 120 μm thick CPDMS strain sensors has highest gauge factor, which was 5.070. In the CPDMS strain sensor durability test, the CPDMS strain sensor could withstand 60 stretch-release cycles, but the resistance would gradually increase. This study provides a method to produce strain sensors cost-effectively. The reproducibility and durability of the sensors, however, requires further investigation in the future.

## Figures and Tables

**Table 1:** The normalized gauge factors.

	No. of Sensor Squares	Gauge Factor	Gauge Factor/No. of Sensor Squares
Grid 5	6	4.396	0.733
Grid 4	5	3.658	0.731
Grid 3	4	3.664	0.916
Grid 2	3	2.838	0.946

## References

[1] Bashir R. (2004). BioMEMS: State-of-the-art in detection, opportunities and prospects. Adv. Drug Deliv. Rev..

[2] Grayson A.R., Shawgo R.S., Johnson A.M., Flynn N.T., Li Y., Cima M.J., Langer R. (2004). A BioMEMS review: MEMS technology for physiologically integrated devices. Proc. IEEE.

[3] Ziaie B., Baldi A., Lei M., Gu Y., Siegel R.A. (2004). Hard and soft micromachining for BioMEMS: Review of techniques and examples of applications in microfluidics and drug delivery. Adv. Drug Deliv. Rev..

[4] Bhushan B. (2007). Nanotribology and nanomechanics of MEMS/NEMS and BioMEMS/BioNEMS materials and devices. Microelectron. Eng..

[5] Yin F., Ye D., Zhu C., Qiu L., Huang Y. (2017). Stretchable, Highly Durable Ternary Nanocomposite Strain Sensor for Structural Health Monitoring of Flexible Aircraft. Sensors.

[6] Zhang S.-H., Wang F.-X., Li J.-J., Peng H.-D., Yan J.-H., Pan G.-B. (2017). Wearable Wide-Range Strain Sensors Based on Ionic Liquids and Monitoring of Human Activities. Sensors.

[7] Mattmann C., Clemens F., Tröster G. (2008). Sensor for measuring strain in textile. Sensors.

[8] Munro B.J., Campbell T.E., Wallace G.G., Steele J.R. (2008). The intelligent knee sleeve: A wearable biofeedback device. Sens. Actuators B Chem..

[9] Scilingo E.P., Lorussi F., Mazzoldi A., De Rossi D. (2003). Strain-sensing fabrics for wearable kinaesthetic-like systems. IEEE Sens. J..

[10] Yamada T., Hayamizu Y., Yamamoto Y., Yomogida Y., Izadi-Najafabadi A., Futaba D.N., Hata K. (2011). A stretchable carbon nanotube strain sensor for human-motion detection. Nat. Nanotechnol..

[11] Chuang H.-S., Wereley S. (2009). Design, fabrication and characterization of a conducting PDMS for microheaters and temperature sensors. J. Micromechan. Microeng..

[12] Lipomi D.J., Vosgueritchian M., Tee B.C., Hellstrom S.L., Lee J.A., Fox C.H., Bao Z. (2011). Skin-like pressure and strain sensors based on transparent elastic films of carbon nanotubes. Nat. Nanotechnol..

[13] Cotton D.P., Graz I.M., Lacour S.P. (2009). A multifunctional capacitive sensor for stretchable electronic skins. IEEE Sens. J..

[14] Mannsfeld S.C., Tee B.C., Stoltenberg R.M., Chen C.V.H., Barman S., Muir B.V., Sokolov A.N., Reese C., Bao Z. (2010). Highly sensitive flexible pressure sensors with microstructured rubber dielectric layers. Nat. Mater..

[15] Mitrakos V., Hands P.J., Cummins G., Macintyre L., Denison F.C., Flynn D., Desmulliez M.P. (2018). Nanocomposite-Based Microstructured Piezoresistive Pressure Sensors for Low-Pressure Measurement Range. Micromachines.

[16] Chang N.-K., Su C.-C., Chang S.-H. (2008). Fabrication of single-walled carbon nanotube flexible strain sensors with high sensitivity. Appl. Phys. Lett..

[17] Cochrane C., Koncar V., Lewandowski M., Dufour C. (2007). Design and development of a flexible strain sensor for textile structures based on a conductive polymer composite. Sensors.

[18] Cai L., Song L., Luan P., Zhang Q., Zhang N., Gao Q., Zhao D., Zhang X., Tu M., Yang F. (2013). Super-stretchable, transparent carbon nanotube-based capacitive strain sensors for human motion detection. Sci. Rep..

[19] Kanoun O., Müller C., Benchirouf A., Sanli A., Dinh T.N., Al-Hamry A., Bu L., Gerlach C., Bouhamed A. (2014). Flexible carbon nanotube films for high performance strain sensors. Sensors.

[20] El Zein A., Huppé C., Cochrane C. (2017). Development of a Flexible Strain Sensor Based on PEDOT: PSS for Thin Film Structures. Sensors.

[21] Wang X., Gu Y., Xiong Z., Cui Z., Zhang T. (2014). Silk-molded flexible, ultrasensitive, and highly stable electronic skin for monitoring human physiological signals. Adv. Mater..

[22] Yogeswaran N., Dang W., Navaraj W.T., Shakthivel D., Khan S., Polat E.O., Cheng G. (2015). New materials and advances in making electronic skin for interactive robots. Adv. Robot..

[23] Bae S.-H., Lee Y., Sharma B.K., Lee H.-J., Kim J.-H., Ahn J.-H. (2013). Graphene-based transparent strain sensor. Carbon.

[24] Kong J.-H., Jang N.-S., Kim S.-H., Kim J.-M. (2014). Simple and rapid micropatterning of conductive carbon composites and its application to elastic strain sensors. Carbon.

[25] Lee C., Jug L., Meng E. (2013). High strain biocompatible polydimethylsiloxane-based conductive graphene and multiwalled carbon nanotube nanocomposite strain sensors. Appl. Phys. Lett..

[26] Liu L., Peng S., Niu X., Wen W. (2006). Microheaters fabricated from a conducting composite. Appl. Phys. Lett..

[27] Lee J., Kim S., Lee J., Yang D., Park B.C., Ryu S., Park I. (2014). A stretchable strain sensor based on a metal nanoparticle thin film for human motion detection. Nanoscale.

[28] Li X., Zhang R., Yu W., Wang K., Wei J., Wu D., Cao A., Li Z., Cheng Y., Zheng Q. (2012). Stretchable and highly sensitive graphene-on-polymer strain sensors. Sci. Rep..

[29] Liu C., Choi J. (2014). Analyzing resistance response of embedded PDMS and carbon nanotubes composite under tensile strain. Microelectron. Eng..

[30] Muth J.T., Vogt D.M., Truby R.L., Mengüç Y., Kolesky D.B., Wood R.J., Lewis J.A. (2014). Embedded 3D printing of strain sensors within highly stretchable elastomers. Adv. Mater..

[31] Shin U.-H., Jeong D.-W., Park S.-M., Kim S.-H., Lee H.W., Kim J.-M. (2014). Highly stretchable conductors and piezocapacitive strain gauges based on simple contact-transfer patterning of carbon nanotube forests. Carbon.

[32] Wang Y., Wang L., Yang T., Li X., Zang X., Zhu M., Wang K., Wu D., Zhu H. (2014). Wearable and highly sensitive graphene strain sensors for human motion monitoring. Adv. Funct. Mater..

[33] Khosla A., Hilbich D., Drewbrook C., Chung D., Gray B.L. (2011). Large scale micropatterning of multi-walled carbon nanotube/polydimethylsiloxane nanocomposite polymer on highly flexible 12 × 24 inch substrates. Micromachining and Microfabrication Process Technology XVI.

[34] Hu N., Fukunaga H., Atobe S., Liu Y., Li J. (2011). Piezoresistive strain sensors made from carbon nanotubes based polymer nanocomposites. Sensors.

[35] Eletskii A.V., Knizhnik A.A., Potapkin B.V., Kenny J.M. (2015). Electrical characteristics of carbon nanotube-doped composites. Phys. Uspekhi.

[36] Khosla A., Gray B.L. (2010). Fabrication of multiwalled carbon nanotube polydimethylsiloxne nanocomposite polymer flexible microelectrodes for microfluidics and MEMS. Electroactive Polymer Actuators and Devices (EAPAD).

[37] Lu N., Lu C., Yang S., Rogers J. (2012). Highly sensitive skin-mountable strain gauges based entirely on elastomers. Adv. Funct. Mater..

[38] Khosla A. (2012). Nanoparticle-doped electrically-conducting polymers for flexible nano-micro Systems. Electrochem. Soc. Interface.

[39] Niu X., Peng S., Liu L., Wen W., Sheng P. (2007). Characterizing and patterning of PDMS-based conducting composites. Adv. Mater..

[40] Lee D., Hong H.P., Lee C.J., Park C.W., Min N.K. (2011). Microfabrication and characterization of spray-coated single-wall carbon nanotube film strain gauges. Nanotechnology.

